# Supplementary material for: Mechanisms of Pseudomonas aeruginosa resistance to type VI secretion system attacks
Source: Nat Commun. 2025 Nov 28;16:10744. doi: 10.1038/s41467-025-65777-x (PMC12663445; doi:10.1038/s41467-025-65777-x)
Supplement: Supplementary file 1 — Supplementary Information [file 41467_2025_65777_MOESM1_ESM.pdf]

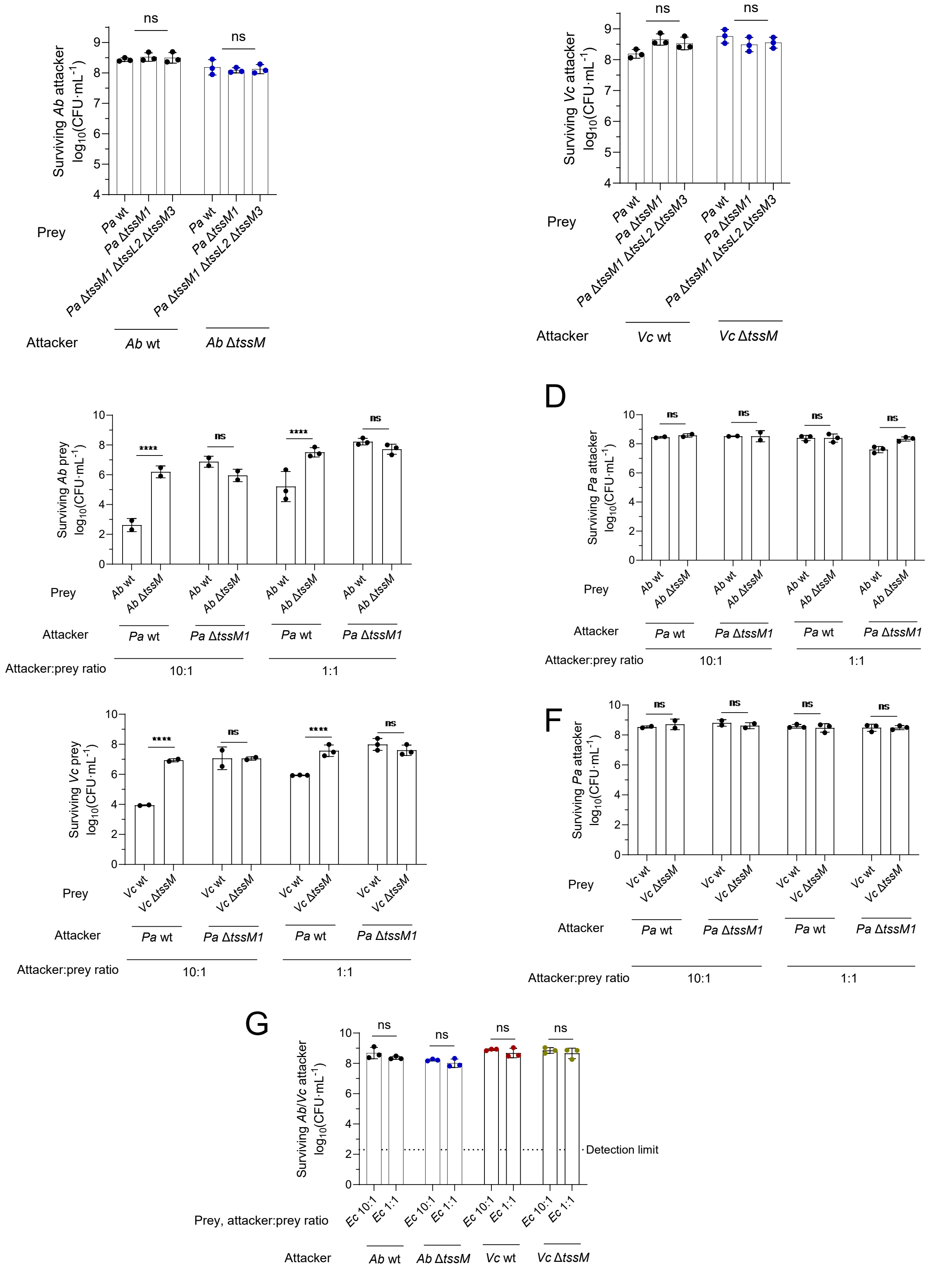

Supplementary Figure 1. *P. aeruginosa* is more resistant to T6SS from *A. baylyi* ADP1 and *V. cholerae* 2740-80 than *E. coli.* CFU counts showing the survival *A. baylyi* (*Ab*) (A) and *V. cholerae* (*Vc*) (B) attacker strains that have or do not have an active T6SS, in a 10:1 attacker:prey ratio where *P.*

aeruginosa is the prey and lacks or not different T6SS clusters. (C) CFU counts showing the survival of prey *A. baylyi* when competed against attacker *P. aeruginosa* in a 10:1 attacker to prey ratio, and (D) survival of the attacker *P. aeruginosa*. Statistics were performed with 2-way ANOVA with Sidak's multiple comparisons correction. **** indicates adjusted p-value <0.0001; ns: non-significant. (E) CFU counts showing the survival of prey *V. cholerae* when competed against attacker *P. aeruginosa* in a 10:1 attacker to prey ratio, and (F) survival of the attacker *P. aeruginosa*. Statistics were performed with 2-way ANOVA with Sidak's multiple comparisons correction. **** indicates adjusted p-value <0.0001; ns: non-significant. (G) CFU counts showing the survival of attacker *A. baylyi* or *V. cholerae* when competed against prey *E. coli* (*Ec*) in 10:1 and 1:1 attacker to prey ratios. Source data are provided as a Source Data file. N=3 biological replicates for all experiments.

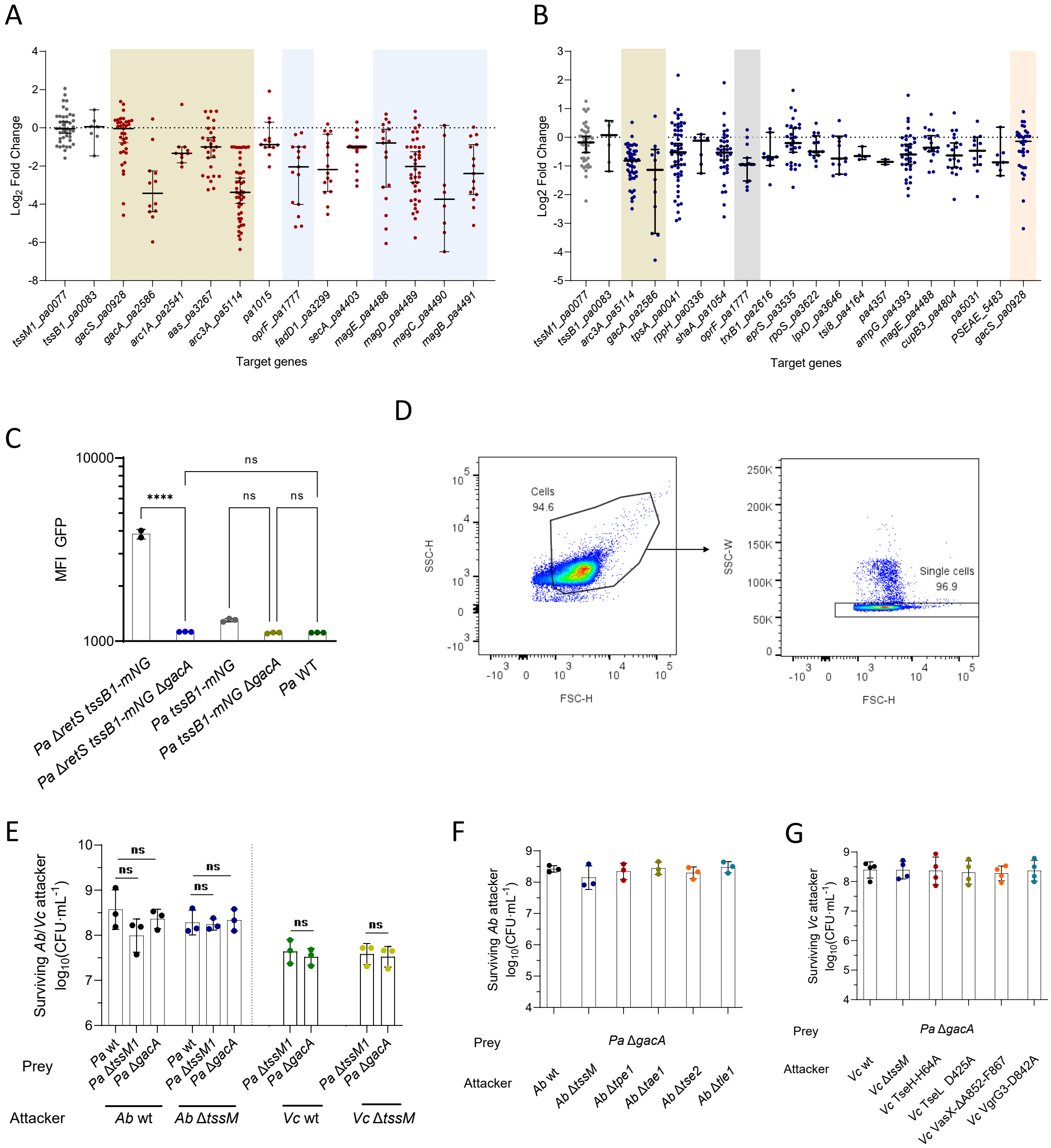

Supplementary Figure 2. Members of the GacA regulon are important for T6SS resistance. Log fold change of each sgRNA targeting the statistically significant hits of the CRISPRi screening where *P. aeruginosa* was competed against *V. cholerae* (A) or *A. baylyi* (B). In (B), one extra non-significant hit (*gacS_pa0928*, shaded in orange) was added, where some sgRNAs displayed a strong depletion whereas others did not. (C) Median Fluorescence Intensity (MFI) of Green Fluorescent Protein (GFP) of wild-type, Δ*retS* and Δ*gacA* strains where the sheath protein TssB1 of the H1-T6SS is labelled with mNeongreen where indicated. TssB1 was used as a proxy for H1-T6SS expression. A strain lacking *retS* displays increased expression of H1-T6SS components and is used here as positive control. 50.000 cells

were analyzed per condition and per replicate. **** indicates p-value of 0.0002 from an un-paired t-test. ns: non-significant (D) Sample gating strategy followed to obtain data from Supplementary Figure 2C. (E) CFU counts showing the survival of attacker *A. baylyi* and *V. cholerae* strains when competed against prey *P. aeruginosa* strains in a 10:1 attacker to prey ratio. Data were analysed with ordinary one-way ANOVA and Dunnett's correction for multiple comparisons. Differences were not significant (adjusted p-value >0.05). (F) CFU counts of attacker *A. baylyi* strains lacking different T6SS effectors when competed against prey *P. aeruginosa* Δ*gacA* in a 10:1 attacker to prey ratio. Data were analysed with ordinary one-way ANOVA and Dunnett's correction for multiple comparisons. Differences were not significant (adjusted p-value >0.05). (G) CFU counts of attacker *V. cholerae* strains carrying different T6SS inactivated effectors when competed against prey *P. aeruginosa* Δ*gacA* in a 10:1 attacker to prey ratio. Data were analysed with ordinary one-way ANOVA and Dunnett's correction for multiple comparisons. Differences were not significant (adjusted p-value >0.05). Source data are provided as a Source Data file. N=3 biological replicates for all experiments.

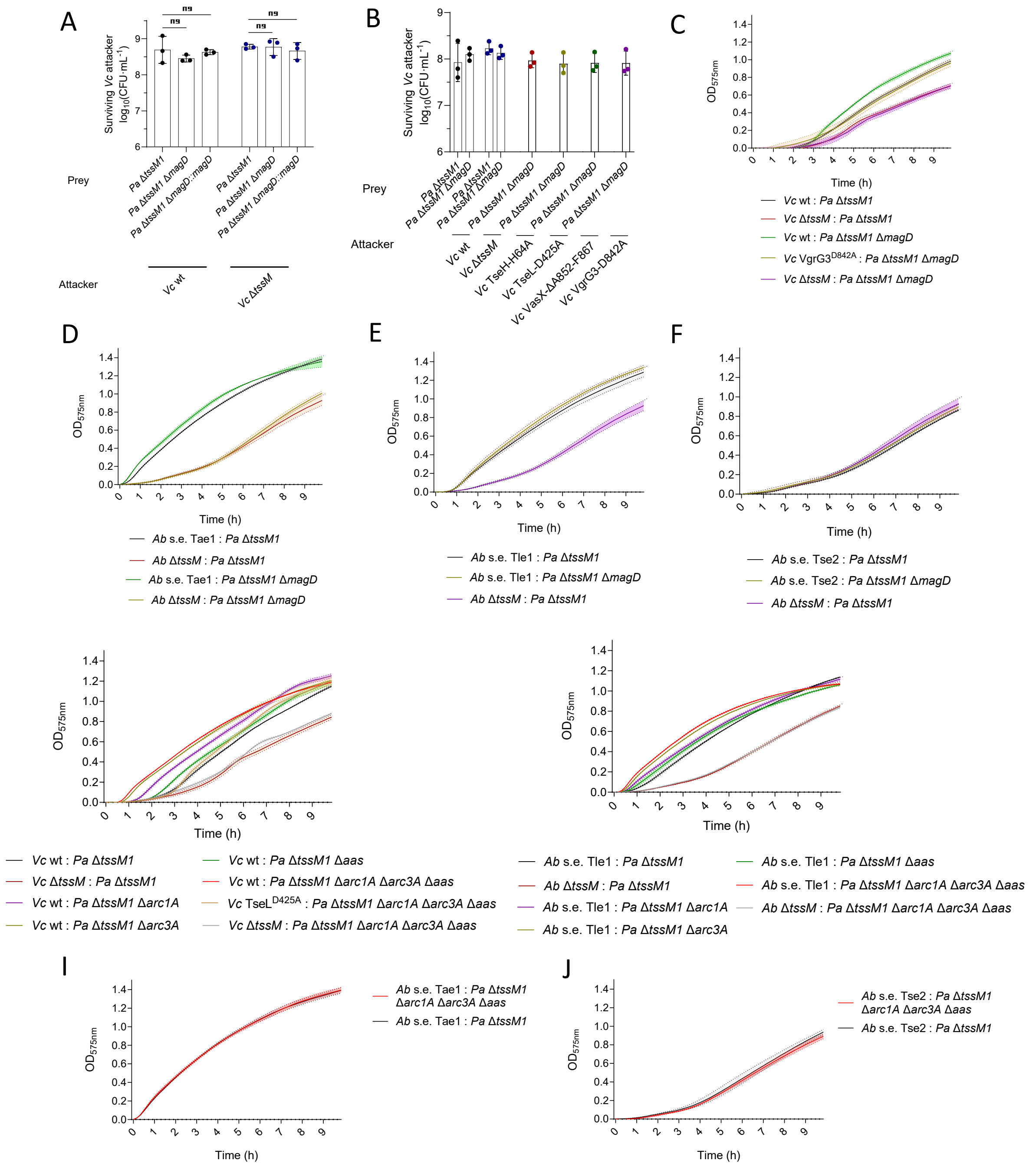

Supplementary Figure 3. MagD is important for resistance against PG-targeting effectors. (A) CFU counts of attacker *V. cholerae* strains with or without an active T6SS when competed against prey *P. aeruginosa* strains with or without *magD* in a 10:1 attacker to prey ratio. Data were analyzed with ordinary two-way ANOVA and Sidak's correction for multiple comparisons. Differences were not statistically significant (adjusted p-value >0.05). (B) CFU counts of attacker *V. cholerae* strains carrying different T6SS inactivated effectors when competed against prey *P. aeruginosa* with or without *magD* in a 10:1 attacker to prey ratio. Data were analyzed with ordinary one-way ANOVA and Dunnett's

correction for multiple comparisons. Differences were not statistically significant (adjusted p-valuo >0.05). CPRG lysis curves of prey, LacZ+ *P. aeruginosa* with or without *magD*, competed against (C) attacker *V. cholerae* carrying or not an active T6SS and an inactivated VgrG3 variant; (D) attacker *A. baylyi* carrying only its PG-targeting effector Tae1; (E) attacker *A. baylyi* carrying only its lipase effector, Tle1; or (F) attacker *A. baylyi* carrying only its Tse2 effector. (G) CPRG lysis curves of prey, LacZ+ *P. aeruginosa* with or without *arc1A*, *arc3A* and *aas*, competed against attacker *V. cholerae* carrying or not an active T6SS and an inactivated variant of its lipase TseL; (H) attacker *A. baylyi* carrying only its lipase effector, Tle1; (I) attacker *A. baylyi* carrying only its PG-targeting effector, Tae1; or (J) attacker *A. baylyi* carrying only its Tse2 effector. All CPRG lysis assays were performed in a 2:1 attacker to prey ratio for 10h at 30ºC. Mean OD_{575nm} is indicated by continuous lines, and the shading around the lines represents the standard deviation. Source data are provided as a Source Data file. N=3 biological replicates for all experiments.

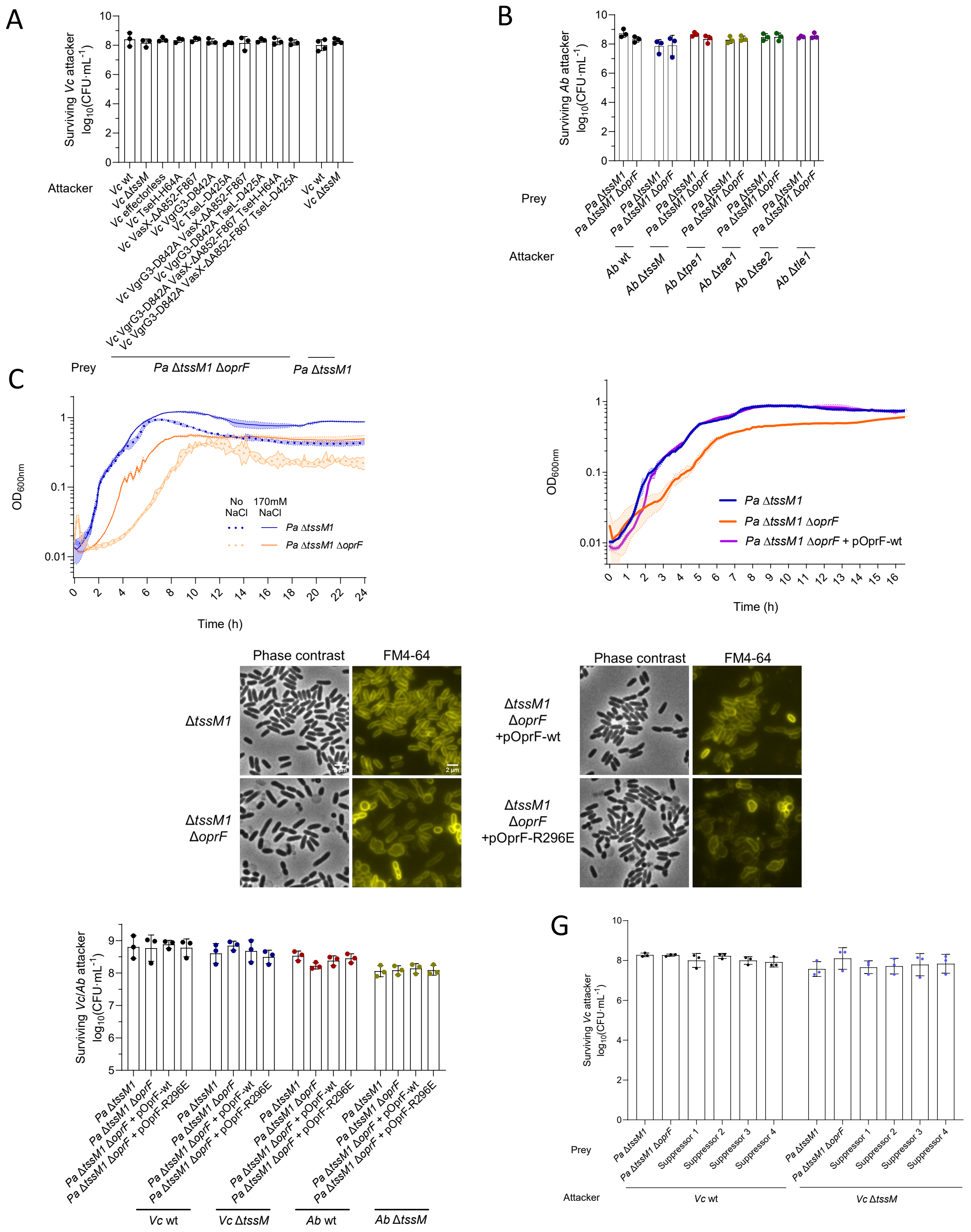

Supplementary Figure 4. **OprF is important for resistance against T6SS attacks.** (A) CFU counts of attacker *V. cholerae* strains carrying different T6SS inactivated effectors when competed against prey *P. aeruginosa* with or without *oprF* in a 10:1 attacker to prey ratio. Data were analysed with ordinary one-way ANOVA and Dunnett's correction for multiple comparisons. Differences were not statistically significant (adjusted p-value >0.05). (B) CFU counts of attacker *A. baylyi* strains lacking different T6SS

when competed against prey *P. aeruginosa* with or without *oprF* in a 10:1 attacker to prey ratio. Data were analysed with ordinary two-way ANOVA and Sidak's correction for multiple comparisons. Differences were not statistically significant (adjusted p-value >0.05). (C) Growth curves of *P. aeruginosa* Δ*tssM1* and Δ*tssM1* Δ*oprF* strains in LB and LB without salt, over 24h at 37ºC. (D) Growth curves of *P. aeruginosa* Δ*tssM1* Δ*oprF* strains complemented *in trans* with wild-type *oprF* in LB without salt over 17h at 37ºC. Mean OD_{600nm} is indicated by continuous lines, and the shading around the lines represents the standard deviation. (E) Fluorescence microscopy images of *P. aeruginosa* lacking *tssM1*, *oprF* and complemented with wild-type or mutant variants of *oprF*. In gray scale, phase contrast channel. In yellow, staining with FM4-64 (membrane dye). (F) CFU counts of attacker *V. cholerae* and *A. baylyi* strains with or without an active T6SS when competed against prey *P. aeruginosa* with different *oprF* variants in a 10:1 attacker to prey ratio. Data were analysed with ordinary one-way ANOVA and Dunnett's correction for multiple comparisons. Differences were not statistically significant (adjusted p-value >0.05). (G) CFU counts of attacker *V. cholerae* when competed against prey *P. aeruginosa* Δ*tssM1* Δ*oprF* suppressors in a 10:1 attacker to prey ratio. Data were analyzed with ordinary two-way ANOVA and Sidak's correction for multiple comparisons. Differences were not statistically significant (adjusted p-value >0.05). Source data are provided as a Source Data file. N=3 biological replicates for all experiments.

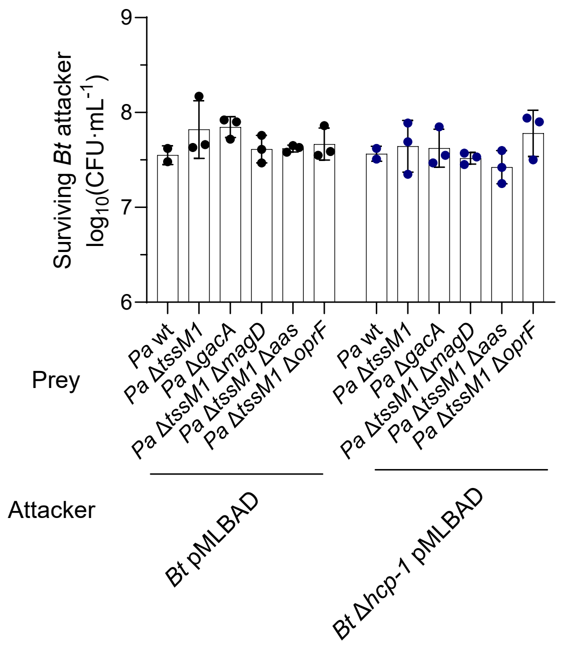

Supplementary Figure 5. T6SS resistance mechanisms are species- or effector-specific. CFU counts of attacker *B. thailandensis* with active or inactive T6SS-1 when competed against prey *P. aeruginosa* strains lacking different T6SS resistance mechanisms in a 10:1 attacker to prey ratio. Data were analyzed with multiple unpaired t tests. Differences were not statistically significant (p-values >0.05). Source data are provided as a Source Data file. N=3 biological replicates.

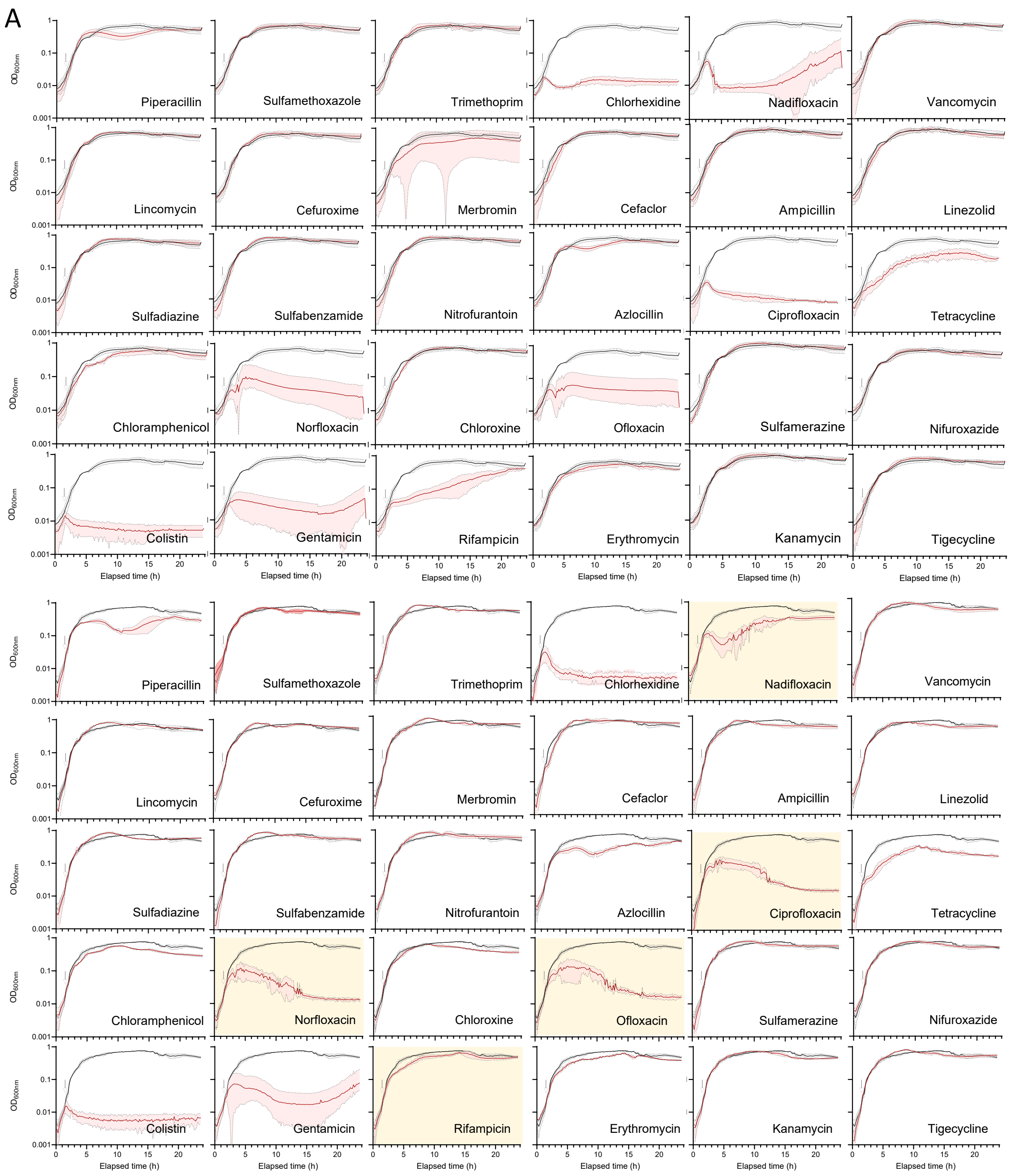

Supplementary Figure 6. T6SS resistance mechanisms influence antibiotic resistance. Growth curves of *P. aeruginosa* strains treated with a DMSO control (black curves) or different antibiotics (red curves) at a concentration of 20 µM. The grey arrow indicates the point at which antibiotics were added. Curves for the (A) wild-type parental strain and (B) Δ*gacA*. The treatments that show different behaviours compared to the parental strain are shaded in yellow. Mean OD_{600nm} is indicated by continuous lines, and the shading around the lines represents the standard deviation. Source data are provided as a Source Data file. N=3 biological replicates.

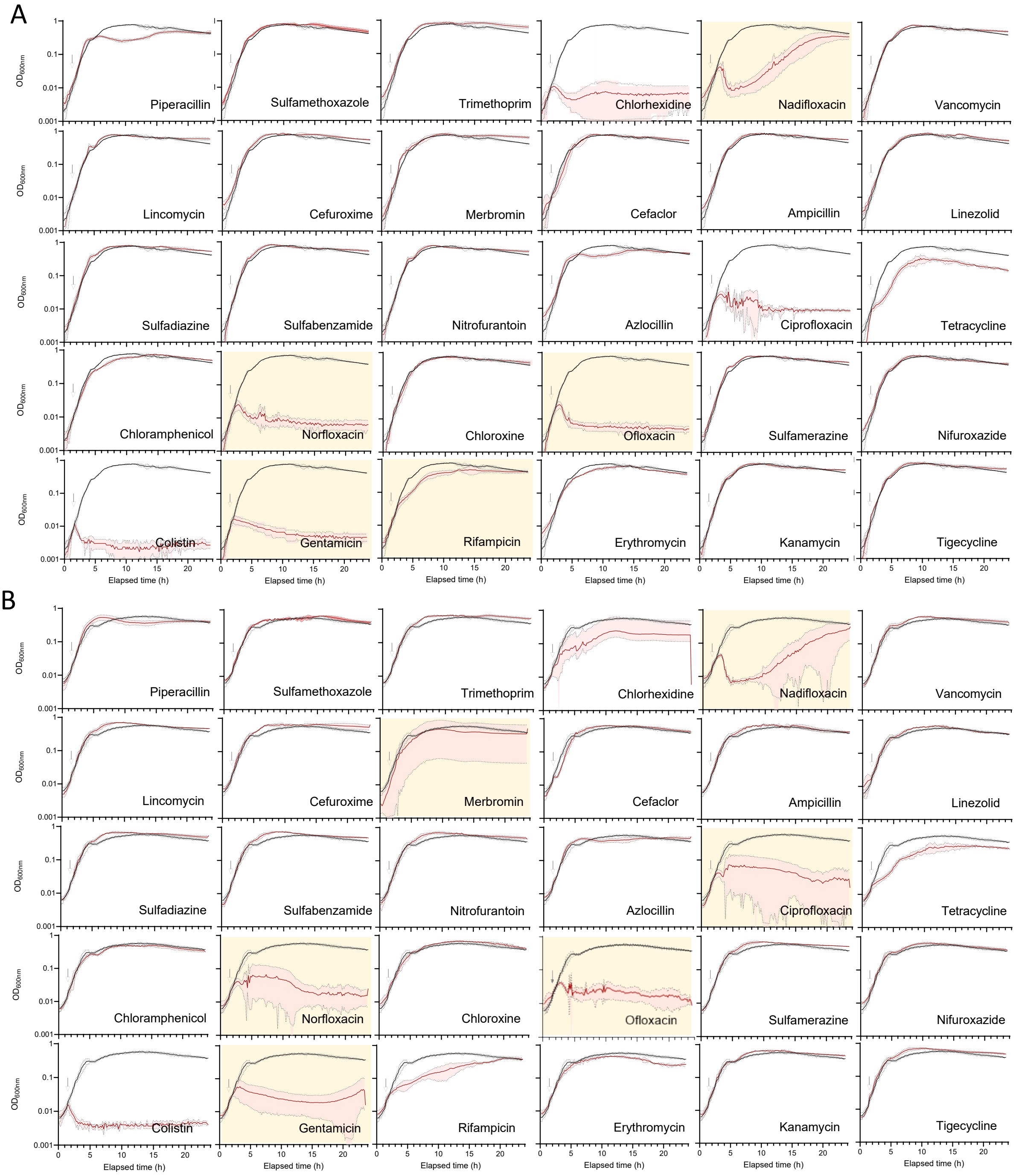

Supplementary Figure 7. T6SS resistance mechanisms influence antibiotic resistance. Growth curves of *P. aeruginosa* strains treated with a DMSO control (black curves) or different antibiotics (red curves) at a concentration of 20 µM. The grey arrow indicates the point at which antibiotics were added. Curves for the (A) Δ*tssM1* and (B) Δ*tssM1* Δ*arc1A* Δ*arc3A* Δ*aas*. The treatments that show different behaviours compared to the parental strain are shaded in yellow. Mean OD_{600nm} is indicated by continuous lines, and the shading around the lines represents the standard deviation. Source data are provided as a Source Data file. N=3 biological replicates.

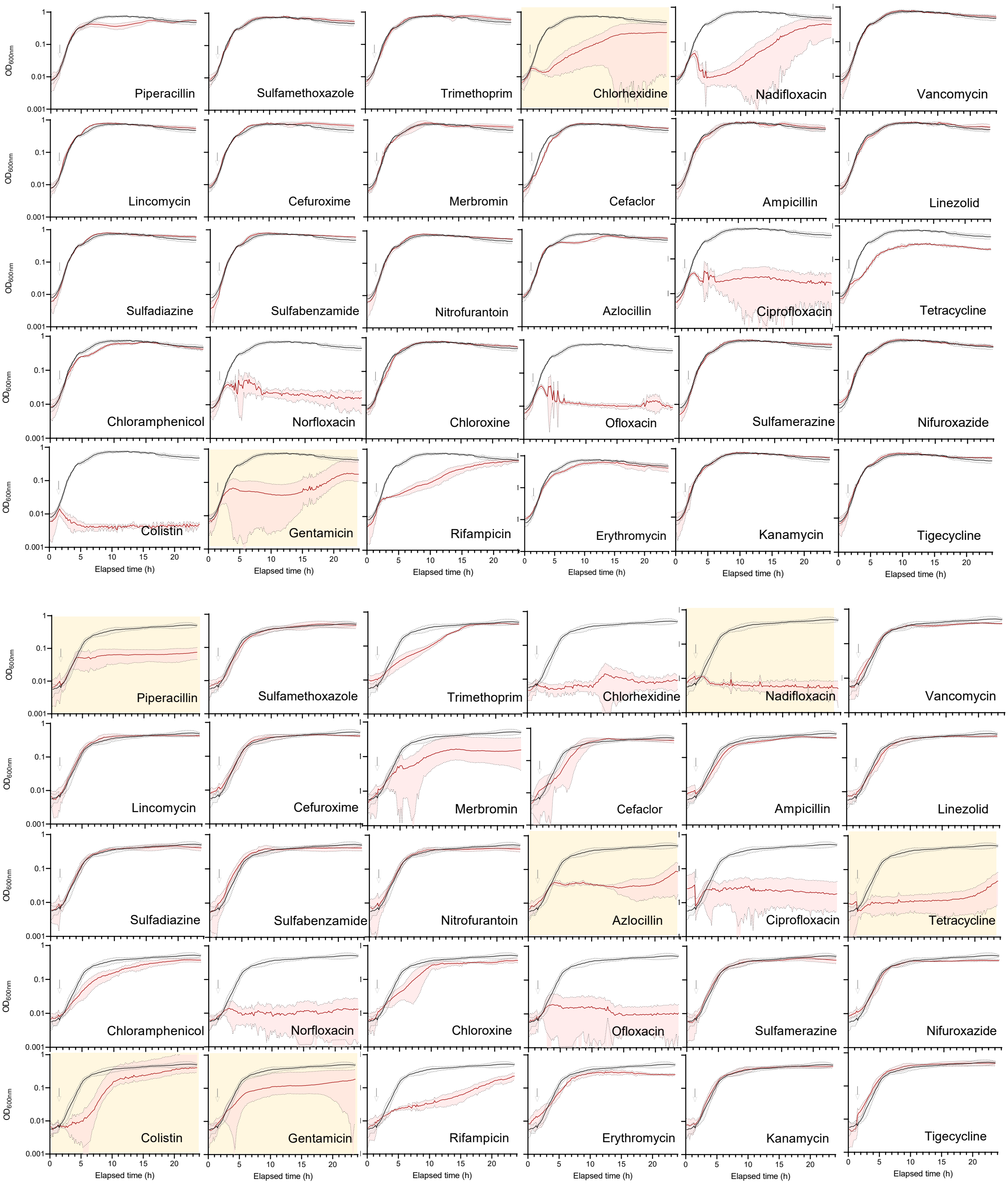

Supplementary Figure 8. T6SS resistance mechanisms influence antibiotic resistance. Growth curves of *P. aeruginosa* strains treated with a DMSO control (black curves) or different antibiotics (red curves) at a concentration of 20 µM. The grey arrow indicates the point at which antibiotics were added. Curves for the (A) Δ*magD* and (B) Δ*oprF* strains. The treatments that show different behaviours compared to the parental strain are shaded in yellow. Mean OD_{600nm} is indicated by continuous lines, and the shading around the lines represents the standard deviation. Source data are provided as a Source Data file. N=3 biological replicates.

Supplementary Table 1. Plasmids used in this study.

Plasmid	Antibiotic resistance	Reference
pEXG2	Gentamicin	[9]
pEXG2-Δ*tssM1*		[5]
pEXG2-Δ*gacA*		This study
pEXG2-Δ*magD*		This study
pEXG2-Δ*aas*		This study
pEXG2-Δ*arc1A*		This study
pEXG2-Δ*arc3A*		This study
pEXG2-Δ*oprF*		This study
pWM91	Ampicillin	[4,10]
pWM91-Δ*tssM*		[11]
pWM91-Δ*tseH*		This study
pWM91-Δ*tseL*		[5]
pWM91-Δ*vasX*		This study
pWM91-Δ*vgrG3*		This study
pWM91-*tseH-H64A*		This study
pWM91-*tseL-D425A*		[5]
pWM91-*vasX-ΔA852-F867*		This study
pWM91-*vgrG3-D842A*		This study
pME3856	Tetracycline	[12]
pPSV35	Gentamicin	[9]
pPSV35-*oprF*		This study
pPSV35-*oprF-R296E*		This study
pMLBAD	Trimethoprim	[13]
pUC18T:TNT7:dCas9Spas	Gentamicin	[14]
pTNS2	Gentamicin	[14]
pFLP2	Carbenicillin	[14]

Supplementary References

1. Taylor, R. G., Walker, D. C. & Mclnnes, R. R. *E.coli* host strains significantly affect the quality of small scale plasmid DNA preparations used for sequencing. *Nucleic Acids Research* **21**, 1677–1678 (1993).

2. Simon, R., Priefer, U. & Pühler, A. A Broad Host Range Mobilization System for In Vivo Genetic Engineering: Transposon Mutagenesis in Gram Negative Bacteria. *Bio/Technology* **1**, 784–791 (1983).

3. Basler, M., Ho, B. T. & Mekalanos, J. J. Tit-for-tat: Type VI secretion system counterattack during bacterial cell-cell interactions. *Cell* **152**, 884–894 (2013).

4. Basler, M., Pilhofer, M., Henderson, G. P., Jensen, G. J. & Mekalanos, J. J. Type VI secretion requires a dynamic contractile phage tail-like structure. *Nature 2012 483:7388* **483**, 182–186 (2012).

5. George, M., Narayanan, S., Tejada-Arranz, A., Plack, A. & Basler, M. Initiation of H1-T6SS dueling between Pseudomonas aeruginosa. *mBio* **15**, (2024).

6. Ringel, P. D., Hu, D. & Basler, M. The Role of Type VI Secretion System Effectors in Target Cell Lysis and Subsequent Horizontal Gene Transfer. *Cell Rep* **21**, 3927–3940 (2017).

7. Stover, C. K. *et al.* Complete genome sequence of Pseudomonas aeruginosa PAO1, an opportunistic pathogen. *Nature* **406**, 959–964 (2000).

8. Ku, J. W. K. *et al.* Bacterial-induced cell fusion is a danger signal triggering cGAS-STING pathway via micronuclei formation. *Proceedings of the National Academy of Sciences of the United States of America* **117**, 15923–15934 (2020).

9. Rietsch, A., Vallet-Gely, I., Dove, S. L. & Mekalanos, J. J. ExsE, a secreted regulator of type III secretion genes in Pseudomonas aeruginosa. *Proc Natl Acad Sci U S A* **102**, 8006–8011 (2005).

10. Metcalf, W. W. *et al.* Conditionally Replicative and Conjugative Plasmids CarryinglacZα for Cloning, Mutagenesis, and Allele Replacement in Bacteria. *Plasmid* **35**, 1–13 (1996).

11. Vettiger, A., Winter, J., Lin, L. & Basler, M. The type VI secretion system sheath assembles at the end distal from the membrane anchor. *Nature Communications 2017 8:1* **8**, 1–9 (2017).

12. Zuber, S. *et al.* GacS sensor domains pertinent to the regulation of exoproduct formation and to the biocontrol potential of Pseudomonas fluorescens CHA0. *Molecular Plant-Microbe Interactions* **16**, 634–644 (2003).

13. Lefebre, M. D. & Valvano, M. A. Construction and evaluation of plasmid vectors optimized for constitutive and regulated gene expression in Burkholderia cepacia complex isolates. *Applied and environmental microbiology* **68**, 5956–5964 (2002).

14. Kaczmarczyk, A., Klotz, A., Manfredi, P. & Jenal, U. Genome-wide high-density CRISPR interference screens reveal condition-specific metabolic vulnerabilities in Pseudomonas aeruginosa PAO1. *bioRxiv* 2025.08.12.669819 (2025) doi:10.1101/2025.08.12.669819.
