# Supplementary material for: Mechanisms of Pseudomonas aeruginosa resistance to type VI secretion system attacks
Source: Nat Commun. 2025 Nov 28;16:10744. doi: 10.1038/s41467-025-65777-x (PMC12663445; doi:10.1038/s41467-025-65777-x)
Supplement: Supplementary file 2 — Description of Additional Supplementary Files [file 41467_2025_65777_MOESM2_ESM.pdf]

Description of Additional Supplementary Files

Supplementary Data 1. Proteomics summary with log2FC, q values and unique peptides detected in the comparison P. aeruginosa ΔgacA versus wild-type. Statistics were calculated using linear mixed-effects models with the MSstats package with default settings.

Supplementary Data 2. Strains used in this study.

Supplementary Data 3. Primers used in this study.
