# Supplementary material for: Mechanisms of Pseudomonas aeruginosa resistance to type VI secretion system attacks
Source: Nat Commun. 2025 Nov 28;16:10744. doi: 10.1038/s41467-025-65777-x (PMC12663445; doi:10.1038/s41467-025-65777-x)
Supplement: Supplementary file 6 — Reporting summary [file 41467_2025_65777_MOESM6_ESM.pdf]

nature portfolio

Corresponding author(s): Marek Basler

Last updated by author(s): Sep 25, 2025

Reporting Summary

Nature Portfolio wishes to improve the reproducibility of the work that we publish. This form provides structure for consistency and transparency in reporting. For further information on Nature Portfolio policies, see our Editorial Policies and the Editorial Policy Checklist.

Statistics

For all statistical analyses, confirm that the following items are present in the figure legend, table legend, main text, or Methods section.

n/a	Confirmed	
☐	☒	The exact sample size (n) for each experimental group/condition, given as a discrete number and unit of measurement
☐	☒	A statement on whether measurements were taken from distinct samples or whether the same sample was measured repeatedly
☐	☒	The statistical test(s) used AND whether they are one- or two-sided<br>*Only common tests should be described solely by name; describe more complex techniques in the Methods section.*
☐	☒	A description of all covariates tested
☐	☒	A description of any assumptions or corrections, such as tests of normality and adjustment for multiple comparisons
☐	☒	A full description of the statistical parameters including central tendency (e.g. means) or other basic estimates (e.g. regression coefficient) AND variation (e.g. standard deviation) or associated estimates of uncertainty (e.g. confidence intervals)
☐	☒	For null hypothesis testing, the test statistic (e.g. F, t, r) with confidence intervals, effect sizes, degrees of freedom and P value noted<br>*Give P values as exact values whenever suitable.*
☒	☐	For Bayesian analysis, information on the choice of priors and Markov chain Monte Carlo settings
☒	☐	For hierarchical and complex designs, identification of the appropriate level for tests and full reporting of outcomes
☒	☐	Estimates of effect sizes (e.g. Cohen's d, Pearson's r), indicating how they were calculated

Our web collection on statistics for biologists contains articles on many of the points above.

Software and code

Policy information about availability of computer code

Data collection	CRISPRi sequencing data was obtained using a Illumina NextSeq 500 sequencer. Proteomics data was obtained using an Orbitrap Exploris 480 mass spectrometer (ThermoFisher Scientific). CPRG lysis assay and growth curve data was collected with an Epoch2 plate reader (BioTek). Flow cytometry data was collected using a BD Fortessa flow cytometer. Fluorescence microscopy data was obtained on a Nikon Ti-E inverted motorized microscope with VisiView software (Visitron Systems). Whole genome sequencing data was obtained with a MinION flow cell.
Data analysis	CRISPRi sequencing data was analyzed with the European Galaxy server using the MAGeCK pipeline as described in materials and methods. Proteomics data was analyzed with SpectroNaut and directDIA. Flow cytometry data was analyzed using FlowJo v10. Imaging data was analyzed using FiJi. Whole genome sequencing data was analyzed with Dorado software and the Evo-genome-analysis Snakemake pipeline. All other analyses were carried out with Prism v 9.3.1.

For manuscripts utilizing custom algorithms or software that are central to the research but not yet described in published literature, software must be made available to editors and reviewers. We strongly encourage code deposition in a community repository (e.g. GitHub). See the Nature Portfolio guidelines for submitting code & software for further information.

Data

Policy information about availability of data

All manuscripts must include a data availability statement. This statement should provide the following information, where applicable:

- Accession codes, unique identifiers, or web links for publicly available datasets
- A description of any restrictions on data availability
- For clinical datasets or third party data, please ensure that the statement adheres to our policy

A data availability statement has been added to the manuscript. Proteomics data can be found at MassIVE under accession number MSV000096798. Sequencing data can be found at ENA under accession number PRJEB84063. Source data can be found in the Source data file and in Zenodo with DOI 10.5281/zenodo.14811406.

Research involving human participants, their data, or biological material

Policy information about studies with human participants or human data. See also policy information about sex, gender (identity/presentation), and sexual orientation and race, ethnicity and racism.

Reporting on sex and gender	N/A
Reporting on race, ethnicity, or other socially relevant groupings	N/A
Population characteristics	N/A
Recruitment	N/A
Ethics oversight	N/A

Note that full information on the approval of the study protocol must also be provided in the manuscript.

Field-specific reporting

Please select the one below that is the best fit for your research. If you are not sure, read the appropriate sections before making your selection.

☒ Life sciences ☐ Behavioural & social sciences ☐ Ecological, evolutionary & environmental sciences

For a reference copy of the document with all sections, see nature.com/documents/nr-reporting-summary-flat.pdf

Life sciences study design

All studies must disclose on these points even when the disclosure is negative.

Sample size	Experiments were performed in biological triplicate.
Data exclusions	No data was excluded from the analysis
Replication	Experimental findings were performed in at least three biological triplicates and found to be reproducible.
Randomization	N/A
Blinding	N/A

Reporting for specific materials, systems and methods

We require information from authors about some types of materials, experimental systems and methods used in many studies. Here, indicate whether each material, system or method listed is relevant to your study. If you are not sure if a list item applies to your research, read the appropriate section before selecting a response.

Materials & experimental systems

n/a	Involved in the study	
☒	☐	Antibodies
☒	☐	Eukaryotic cell lines
☒	☐	Palaeontology and archaeology
☒	☐	Animals and other organisms
☒	☐	Clinical data
☒	☐	Dual use research of concern
☒	☐	Plants

Methods

n/a	Involved in the study	
☒	☐	ChIP-seq
☐	☒	Flow cytometry
☒	☐	MRI-based neuroimaging

Plants

Seed stocks	N/A
Novel plant genotypes	N/A
Authentication	N/A

Flow Cytometry

Plots

Confirm that:

☒ The axis labels state the marker and fluorochrome used (e.g. CD4-FITC).

☒ The axis scales are clearly visible. Include numbers along axes only for bottom left plot of group (a 'group' is an analysis of identical markers).

☐ All plots are contour plots with outliers or pseudocolor plots.

☒ A numerical value for number of cells or percentage (with statistics) is provided.

Methodology

Sample preparation	Bacteria were grown to mid-exponential phase in LB. They were diluted 1.100 in PBS and measured in the flow cytometer.
Instrument	FACS Analyzer LSR Fortessa (BD)
Software	Collection in BD FACSDiva Software. Analysis in FlowJo v10.
Cell population abundance	Single cell bacteria represented >95% of the measured events.
Gating strategy	FSC and SSC gating was performed to remove electronic noise and all larger events were included in the analysis. Single cells were gated by selecting SSC-W values smaller than 70k.

☒ Tick this box to confirm that a figure exemplifying the gating strategy is provided in the Supplementary Information.
